# Aerobic exercise ameliorates atherosclerosis-induced cognitive impairment via hippocampal IL-33/NF-κB signaling modulation

**DOI:** 10.3389/fphys.2025.1608268

**Published:** 2025-06-10

**Authors:** Jianwei Peng, Zaoshang Chang, Jinyun Wang, Zijie Liao, Yunjie Yang, Lianwei Mu, Shen Wang, Junhao Huang, Jingbo Xia, Min Hu

**Affiliations:** ^1^ Guangdong Provincial Key Laboratory of Physical Activity and Health Promotion, Guangzhou Sport University, Guangzhou, China; ^2^ Department of Physiology, Shaoyang University, Shaoyang, China

**Keywords:** aerobic exercise, atherosclerosis, cognitive impairment, IL-33 signaling, inflammatory cytokines

## Abstract

Atherosclerosis (AS), a common cardiovascular condition, is often linked to cognitive dysfunction. This study investigates how aerobic exercise affects cognitive impairments caused by AS in ApoE^−/−^ mice. At 8 weeks old, male ApoE^−/−^ mice were fed a high-fat, high-cholesterol diet (HFHCD) for 6 weeks to induce AS, with C57BL/6J mice on a standard diet as control group (WT). Initially, the study compared aortic plaque and cognitive performance between the WT and AS mice. Then, AS mice were divided into sedentary (AS-SED) and exercise (AS-EX) groups for a 10-week aerobic exercise intervention. Results showed significant aortic plaques and cognitive deficits in AS mice after 6 weeks on the HFHCD diet. However, the 10-week exercise reduced plaque, improved cognition, and enhanced cerebral blood flow. Exercise intervention also decreased IL-33 expression in the hippocampus and inhibited NF-κB and IκBα phosphorylation. Furthermore, aerobic exercise reduces M1 microglial activation and pro-inflammatory cytokines like IL-6, TNF-α, and IL-1β in the hippocampus of AS mice, thereby decreasing neuroinflammation. In summary, aerobic exercise can effectively improve cognitive function by decreasing IL-33 expression and inhibiting NF-κB activation, which in turn reduces microglial activation and the release of inflammatory mediators in the hippocampus. This study provides evidence for aerobic exercise to improve cognitive impairment caused by AS.

## 1 Introduction

Atherosclerosis (AS) is a prevalent form of cardiovascular disease, primarily characterized by lipid accumulation and inflammation within the large arteries. This condition can ultimately result in clinical complications such as myocardial infarction and stroke (Björkegren and Lusis, 2022). Research has demonstrated a correlation between AS and cognitive decline (Cortes-Canteli et al., 2021). Among individuals with coronary heart disease attributable to AS, the prevalence of cognitive impairment ranges from 35% to 53% (Newman et al., 2001). The mechanisms through which AS contributes to cognitive impairment are likely multifaceted. Specifically, AS in regions such as the aorta and coronary arteries may lead to diminished cerebral blood flow, increased microvascular damage, heightened blood-brain barrier permeability, exacerbated inflammatory responses and oxidative stress, and the development of white matter lesions. These pathological changes in brain structure and function can subsequently impair synaptic plasticity and cognitive function (Denes et al., 2012; Alber et al., 2019; Kiss et al., 2019; Li B. et al., 2019; Nyúl-Tóth et al., 2024).

The positive effects of exercise on the cardiovascular system are well-documented, with consistent engagement in aerobic or strength training shown to decrease both the incidence and mortality rates associated with cardiovascular diseases (Lee et al., 2014). Furthermore, aerobic exercise is extensively acknowledged for its capacity to enhance cognitive function and has been a central subject of investigation concerning the impact of physical activity on cognitive decline related to aging (Laurin et al., 2001; García-Mesa et al., 2011). Prior research has predominantly focused on the role of aerobic exercise in preventing the onset and progression of AS through various mechanisms, such as improved metabolism, reduced inflammation, and enhanced vascular function (Goldberg, 1989; Liu et al., 2023). Nonetheless, there is a paucity of research investigating exercise-mediated mechanisms specifically targeting AS-induced cognitive dysfunction. The molecular pathways mediating exercise-induced AS improvement may not necessarily translate to its complicating disease of cognitive dysfunction. Consequently, this study prioritizes hippocampal pathway analysis to elucidate exercise-induced neuroprotection in AS-related cognitive impairment.

To validate the experimental objectives, an AS model of cognitive impairment was established in ApoE^−/−^ mice through the administration of a high-fat, high-cholesterol diet (HFHCD). Subsequently, ApoE^−/−^ mice exhibiting cognitive impairment underwent a 10-week aerobic exercise regimen to assess whether this intervention could reduce aortic plaque area and alleviate cognitive deficits in the AS model. Should aerobic exercise demonstrate cognitive improvements in the AS model, further investigations will be conducted to elucidate the underlying mechanisms of cognitive enhancement.

## 2 Materials and methods

### 2.1 Animals

The study was conducted in accordance with the Declaration of Helsinki, and approved by the by the Animal Experimental Ethics Inspection of Guangzhou Sport University (protocol code 2024DWLL-38 and 10 July 2024 of approval). Healthy 8-week-old male C57BL/6J (n = 11) and ApoE^−/−^ mice (n = 22) were purchased from Cyagen Biotechnology Co., Ltd (Suzhou, China). All mice were housed and trained in a specific pathogen-free (SPF) laboratory animal facility at the Guangzhou sport university science experiment center, with *ad libitum* access to water and maintained on a 12-h light/dark cycle. Following the experiments, the mice were euthanized via cervical dislocation under 2.5% isoflurane anesthesia.

### 2.2 Experimental design

**FIGURE 1:**
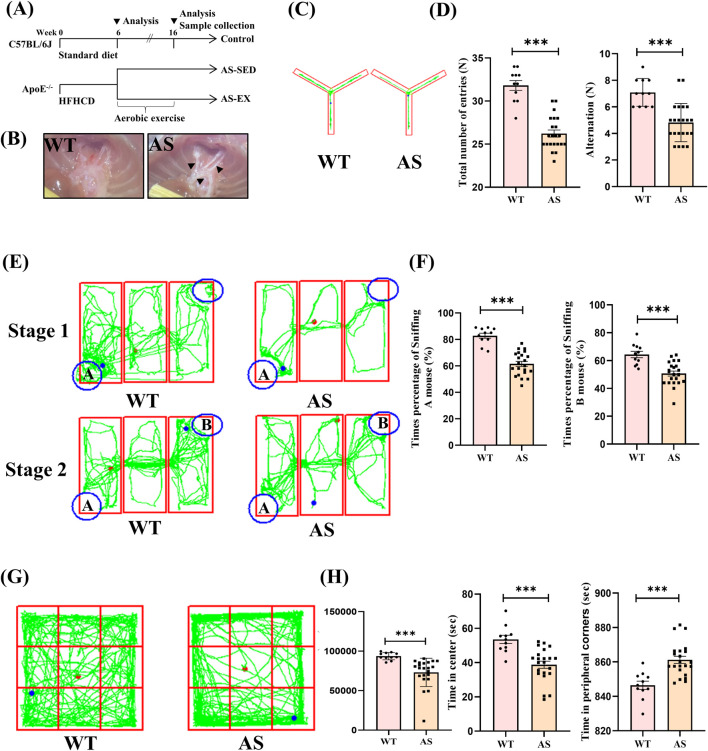
6-week high-fat high-cholesterol diet (HFHCD) induces increased aortic atherosclerotic plaque area and cognitive impairment in ApoE^−/−^ mice. **(A)** Overall experimental design process and grouping. **(B)** Microscopic images of aortic plaques in AS and WT mice. **(C and D)** Y-maze trajectory plots and quantification of total number of entries and alternation counts in WT (n = 11) and AS (n = 22) mice. **(E)**–**(F)** Three-chamber social test trajectory plots and percentage of sniffing times for mice A and B in WT (n = 11) and AS (n = 22) mice. **(G)**–**(H)** Open-field test trajectory plots and quantification of total distance moved, time spent in the center area, and time spent in the peripheral corners in WT (n = 11) and AS (n = 22) mice. Data are presented as Mean ± SEM, ***P < 0.001.

In this study, ApoE^−/−^ mice subjected to HFHCD were designated as AS group, whereas C57BL/6J mice maintained on a standard diet served as the wild-type (WT) control group. After a 6-week feeding period, initial cognitive function assessments were conducted for both groups, utilizing the Y-maze, open-field, and three-chamber social tests. Subsequently, three mice from each group were randomly selected for dissection. Aortic atherosclerotic plaques were examined using optical microscopy. The remaining mice (n = 16) in the AS group were randomly allocated into AS-SED and AS-EX subgroups (n = 8 per group), while continuing their original diet, and the WT group persisted on the standard feed (n = 8). The AS-EX subgroup participated in a 10-week aerobic exercise intervention, with the AS-SED and WT groups serving as sedentary controls (Figure 1A). Upon completion of the 10-week treadmill aerobic exercise intervention, behavioral assessments were repeated, followed by cerebral blood flow evaluations. Subsequently, the aortas were excised for Oil Red O staining to evaluate plaque area, and the complete brains along with the hippocampi were collected from mice across all three experimental groups.

### 2.3 Exercise intervention protocol

Building upon previous research and made modifications (Pynn et al., 2004), this experiment implemented a moderate intensity aerobic exercise protocol using a zero-degree incline treadmill. The mice were subjected to exercise at a speed of 14 m/min. Considering that the subjects involved in the experiment were atherosclerotic model mice, we arranged for them to rest for 2 min after every 15 min of exercise. This cycle was repeated 4 times, resulting in a total exercise duration of 68 min per session. The exercise was scheduled for 5 days per week over a period of 10 weeks.

### 2.4 Y-maze test

The experiment was performed using a Y-shaped maze. Each mouse was positioned at the starting point of one arm of the maze and permitted to explore freely for a duration of 8 min. The parameters evaluated included: (1) the total number of arm entries, and (2) the count of spontaneous alternations. Locomotor activity was quantified by the total number of arm entries, whereas the number of spontaneous alternations served as an indicator of the mice’s short-term memory capacity (Farkas et al., 2022).

### 2.5 Open-field test

The experiment is performed in an open square arena, where the subject animal is positioned at the center, and its spontaneous activity is monitored using a video tracking system over a duration of 15 min. The primary parameters measured during the test include the total distance traveled, the duration of time spent in the central area, and the duration of time spent in the peripheral corners. These metrics are instrumental in assessing the animal’s exploratory behavior, anxiety levels, and motor capabilities (Wang et al., 2023).

### 2.6 Three-chamber social test

The three-chamber social test is primarily used to assess the social capabilities, social memory, and preference for novelty in mice (Kaidanovich-Beilin et al., 2011). The experimental setup consists of three transparent rectangular chambers. The experiment is divided into two phases:

Phase 1: A novel mouse (A) is placed in a circular wire cage on one side, while the wire cage on the opposite side remains unoccupied. At the commencement of this phase, the test mice are introduced from the central chamber and permitted to freely explore all three chambers for a duration of 10 min. Upon the conclusion of this phase, the three chambers are disinfected with alcohol to remove any residual odors left by the mice. Phase 2: A second novel mouse (B) is introduced into the previously unoccupied wire cage, and the test mice are once again introduced from the central chamber to explore the three chambers for an additional 10 min. During the experiment, an interaction with the wire cage is deemed a valid investigative behavior when the test mice make direct contact with the cage or remain within a 3–5 cm proximity to it. The following metrics are calculated: the frequency of sniffing behaviors directed towards mice A and B, expressed as a percentage of total interactions.

### 2.7 Laser speckle contrast imaging (LSCI) for cerebral blood flow measurement

Effective anesthesia is initially achieved through isoflurane inhalation, after which the mice are securely positioned on a specially designed experimental board. The hair on the mice’s heads is subsequently removed using depilatory cream, followed by a careful incision of the scalp to fully expose the skull. Any residual hair and debris on the skull surface are meticulously cleaned using cotton swabs moistened with distilled water. Following this preparatory procedure, LSCI is employed (Simopto, China), wherein a laser is directly applied to the surface of the mice’s skull, and a continuous series of 30 speckle images is captured for each subject.

### 2.8 Animal tissue collection and processing

Following the measurement of cerebral blood flow, the thoracic cavity of the mouse was accessed to expose the heart. A perfusion needle was inserted into the apex of the heart, and a swift incision was made in the right auricular region. Saline perfusion was initiated, and upon observing a lightening of the liver color, 4% paraformaldehyde (PFA) was introduced until the limbs exhibited rigidity. Subsequently, the aorta and intact brain tissue were excised. The intact brain tissue was then immersed in 4% PFA fixative for a duration of 24 h to ensure adequate tissue fixation. In parallel, hippocampal tissue from the remaining mice was isolated and promptly placed into sterile centrifuge tubes containing RNA preservation buffer to preserve RNA integrity for subsequent transcriptome sequencing.

### 2.9 Aortic Oil Red O staining

The excised aortas were fixed in 4% PFA for a duration of 24 h. Following fixation, the specimens were rinsed three times with phosphate-buffered saline (PBS), with each wash lasting 3 min. Subsequently, the aortic specimens underwent incubation in an Oil Red O staining solution at 40°C for a period of 1 h. Post-staining, the samples were washed twice with 75% ethanol. During the differentiation process, the samples were rinsed twice with distilled water to eliminate any residual ethanol. Finally, the stained aortic samples were promptly observed and photographed to document and preserve the experimental results.

### 2.10 RNA sequencing

The hippocampal tissues of mice in the AS-SED and AS-EX groups were used to extract total RNA, and four samples from each group were analyzed using RNA sequencing. The RNA sequencing and subsequent data analysis were conducted by Applied Protein Technology Co., Ltd.

### 2.11 qRT-PCR

In this experiment, total RNA was extracted from hippocampal tissue using the Trizol reagent (Magen, China). Reverse transcription was performed on the extracted RNA using the RT SuperMIX for RT-qPCR kit (G3337, Servicebio, China) to synthesize the corresponding cDNA. PCR was conducted using the SGExcel FastSYBR RT-qPCR Premix Kit (B532955, Sangon biotech, China). The primer sequences are listed in Table 1.

**TABLE 1:** Primer list.

Gene	Primer Sequences (5′to 3′)
Ccl17-F	TACCATGAGGTCACTTCAGATGC
Ccl17-R	GCACTCTCGGCCTACATTGG
Muc5b-F	GTGGCCTTGCTCATGGTGT
Muc5b-R	GGACGAAGGTGACATGCCT
IL-33-F	CCTGGCTCTTGCTTGCCTT
IL-33-R	GGTCTTGTGTGATGTTGCTCA
Edn1-F	GCACCGGAGCTGAGAATGG
Edn1-R	GTGGCAGAAGTAGACACACTC
Hcar1-F	TCGTGCTGTCTCATCGAGG
Hcar1-R	TCTTCATGTGAAAGCAGAAGCC
Il1-β -F	GCAACTGTTCCTGAACTCAACT
Il1-β-R	ATCTTTTGGGGTCCGTCAACT
Il-6 -F	TAGTCCTTCCTACCCCAATTTCC
Il-6-R	TTGGTCCTTAGCCACTCCTTC
TNF-α-F	CCCTCACACTCAGATCATCTTCT
TNF-α-R	GCTACGACGTGGGCTACAG
GAPDH-F	TGTGTCCGTCGTGGATCTGA
GAPDH-R	CCTGCTTCACCACCTTCTTGA

The ABI StepOne Plus cycler, along with StepOne software, was used for amplification and detection. The qPCR procedure began with a 3-min step at 95°C, then proceeded with 40 cycles of 5 s at 95°C and 20 s at 60°C. GAPDH was used as the internal standard control to normalize gene expression using the 2^−ΔΔCt^ method (Yang et al., 2022).

### 2.12 Immunofluorescence staining

Following a 24-h fixation period in 4% PFA, the brain tissues underwent dehydration through a gradient series of sucrose solutions with concentrations of 15%, 20%, and 30%, each for 24 h. Subsequently, the tissues were cryopreserved in OCT embedding medium (Servicebio, China) and sectioned into 16 μm continuous coronal slices using a cryostat microtome (Leica Microsystems, Germany). The primary antibodies utilized included anti-Iba1 antibody (1:500, GB12105, Servicebio, China), anti-CD68 antibody (1:200, D194559, Sangon Biotech), and anti-IL-33 antibody (1:100, 12372-1-AP, Proteintech, China). The secondary antibodies employed were Alexa Fluor 488-conjugated goat anti-mouse IgG fluorescent secondary antibody (green, 1:400, GB25301, Servicebio, China), CY3-conjugated goat anti-mouse IgG fluorescent secondary antibody (red, 1:300, GB21301, Servicebio, China), along with DAPI staining solution (G1012, Servicebio, China). The examination and imaging were performed using an Olympus confocal microscope (OLYMPUS FV31S-SW, Japan).

### 2.13 Western blotting (WB)

Hippocampal tissues from mice were lysed utilizing a lysis buffer (P0013B, Beyotime Biotechnology). Protein concentrations were subsequently determined for Western blot (WB) analysis. The primary antibodies employed included anti-IL-33 antibody (1:1000, 12372-1-AP, Proteintech, China), anti-IκB alpha (18220-1-AP, Proteintech, China), anti-phospho-IκB alpha (68999-1-Ig, Proteintech, China), anti-NF-κB (GB11997, Servicebio, China), anti-phospho-NF-κB (GB113882, Servicebio, China), and anti-Gapdh (GB12002, Servicebio, China). The secondary antibody used was anti-Rabbit IgG (GB111738, Servicebio, China). Image-Pro Plus version 6.0 was used to quantify the chemiluminescent signal.

### 2.14 Statistical analysis

Data analysis was performed with GraphPad Prism 9.5.1 software. Each experiment or group’s sample number (n) is noted in the figure legends. Among three groups, statistical analysis was performed using one-way ANOVA with Tukey’s multiple comparisons test. Comparisons between two groups were analyzed using unpaired and two-tailed Student’s t-test. All data are presented as the mean ± SEM. Video tracking system (shxinruan, China) for recording and analyzing behavioral tests. Values of *P* < 0.05 were considered statistically significant.

## 3 Results

### 3.1 6 weeks of HFHCD induced atherosclerotic plaque area enlargement in the aortas of ApoE^−/−^ mice and led to cognitive impairments

The experimental design and grouping are shown in (Figure 1A). Macroscopic examination of the aorta demonstrated that 6 weeks on HFHCD (pre-intervention modeling phase) resulted in the formation of significant atherosclerotic plaques in the AS group, whereas the WT group, which was fed a standard diet, did not develop such plaques. This finding initially confirmed the successful establishment of the atherosclerotic model in the AS mice (Figure 1B). In the Y-maze test (Figures 1C,D), both the total number of entries and the number of alternations made by the AS group were significantly lower than those of the WT group, indicating impairments in spatial memory and decision-making abilities in the AS mice. Furthermore, results from the three-chamber social test revealed deficits in social behavior in the AS mice (Figures 1E,F). During both the first and second phases of the test, the WT group mice exhibited a significantly higher percentage of sniffing interactions with mice A and B compared to the AS group mice, suggesting that AS may adversely affect the social cognitive function of AS mice. In conclusion, the open-field test showed that AS group mice traveled significantly less distance than WT group mice, suggesting reduced spontaneous activity in the AS mice. Furthermore, the AS group mice exhibited a marked decrease in time spent in the central area and an increase in time spent in the peripheral corners. This behavior may suggest heightened anxiety levels, as exploration of the central area is generally associated with lower anxiety, whereas exploration of the peripheral corners is linked to higher anxiety (Figures 1G,H). These findings indicate that 6 weeks of HFHCD resulted in an increased atherosclerotic plaque area in ApoE^−/−^ mice and the onset of cognitive impairments. Thus, mice with atherosclerotic pathology and differences in cognitive function meet criteria for subsequent experiments before proceeding with exercise intervention.

### 3.2 Aerobic exercise reduces aortic plaque area and improves cognition and cerebral blood flow in AS mice

**FIGURE 2:**
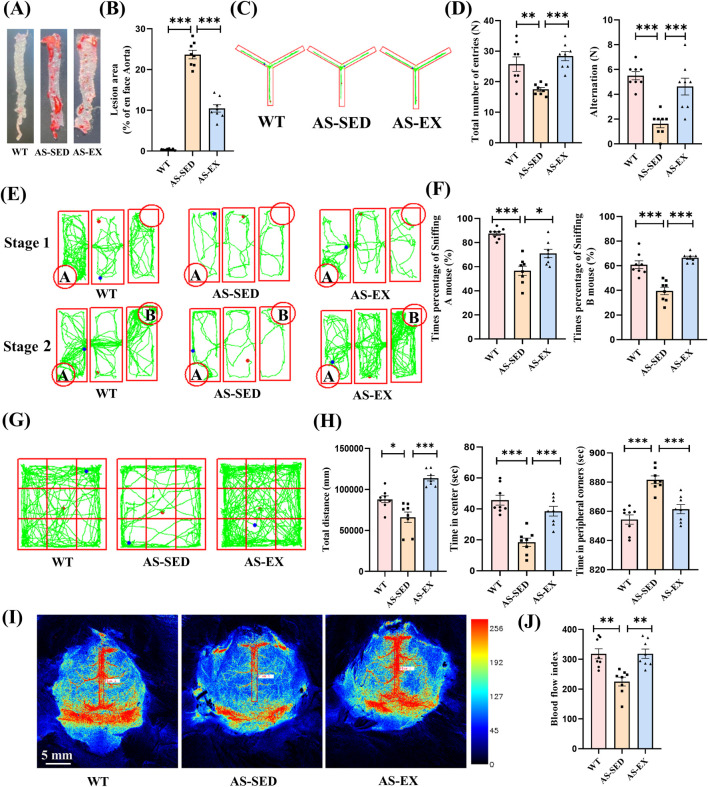
Aerobic exercise reduces aortic plaque area, improves cognitive function, and enhances cerebral blood flow in AS mice. **(A)**–**(B)** Aortic Oil Red O staining images of mice from three groups and their statistical results (n = 8 per group). **(C)**–**(D)** Y-maze trajectory plots and quantification of total number of entries and alternation counts in the three groups (n = 8 per group). **(E)**–**(F)** Three-chamber social test trajectory plots and percentage of sniffing times for mice A and B in the three groups (n = 8 per group). **(G)**–**(H)** Open-field test trajectory plots and quantification of total distance moved, time spent in the center area, and time spent in the peripheral corners in the three groups (n = 8 per group). **(I)**–**(J)** Pseudo color images of laser speckle cerebral blood flow imaging and blood flow index statistical results at the sagittal suture site of the brain for the three groups of mice (n = 8 per group). Data are shown as Mean ± SEM, *P < 0.05, **P < 0.01, ***P < 0.001.

The AS-EX group showed a notable decrease in atherosclerotic plaque area as indicated by Aortic Oil Red O staining, compared to the AS-SED group (Figures 2A,B). Following the exercise intervention, the Y-maze test (Figures 2C,D) demonstrated that the AS-EX group exhibited a significantly higher number of total entries and alternations relative to the AS-SED group, suggesting that exercise improved spatial memory and decision-making abilities in AS mice. The results from the three-chamber social test further corroborated the positive effects of exercise intervention on the social cognition of AS mice (Figures 2E,F). During the initial phase of the test, the AS-EX group spent a significantly greater proportion of times sniffing mouse A compared to the AS-SED group. In the subsequent phase, the AS-EX group also spent a significantly greater proportion of times sniffing mouse B compared to the AS-SED group. Lastly, the open field test indicated a significant increase in the total distance traveled by the AS-EX group compared to the AS-SED group, suggesting that exercise intervention substantially enhanced the spontaneous activity of AS mice. Furthermore, the AS-EX group exhibited a marked increase in the duration spent in the central area and a reduction in time spent in the peripheral corners relative to the AS-SED group, indicating an amelioration of anxiety levels in AS mice following the exercise intervention (Figures 2G,H). Cerebral blood flow was assessed in mice via the sagittal suture to provide an indication of global cerebral perfusion (Figure 2I). The blood flow index in the AS-SED group was significantly lower than that observed in the WT group, whereas the AS-EX group demonstrated a significant enhancement in cerebral blood flow compared to the AS-SED group (Figure 2J). These findings suggest that exercise facilitates improvements in cerebral blood flow in AS mice.

### 3.3 Aerobic exercise induces changes in genes within the hippocampal tissue of mice with cognitive impairment induced by AS

**FIGURE 3:**
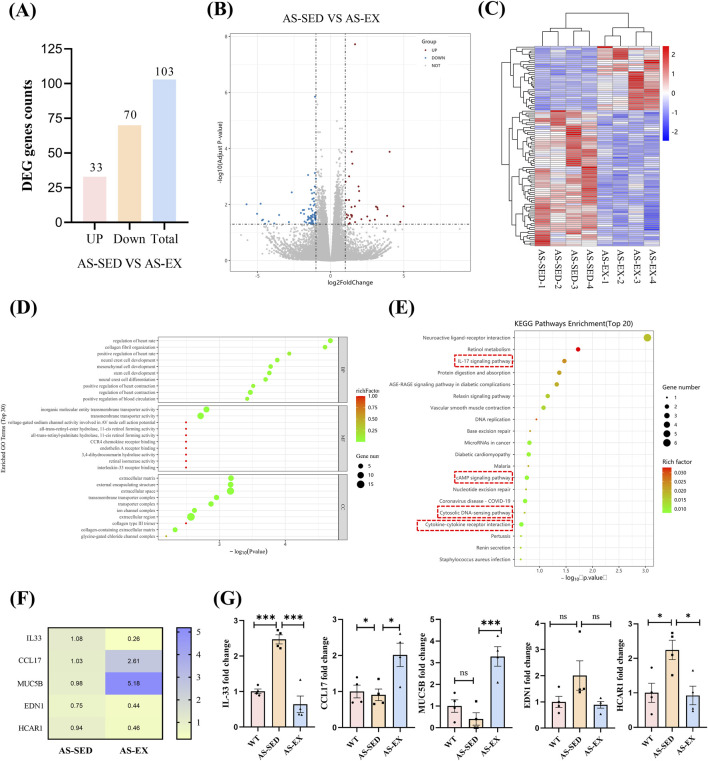
RNA-Seq results and target gene screening. **(A)** Bar chart of differentially expressed genes. **(B)** Volcano plot of differential gene expression distribution. **(C)** Heatmap of differential gene clustering. **(D)** Bubble chart of GO enrichment for differential genes. **(E)** Bubble chart of KEGG enrichment for differentially expressed genes. **(F)** Heatmap of the normalized expression of IL-33, CCL17, MUC5B, EDN1, and HCAR1 genes from the RNA-Seq data. **(G)** qRT-PCR results in the hippocampus of mice three groups (n = 4 per group). The screening criteria for differentially expressed genes were |log2 (FoldChange)| > 1 & padj ≤0.05. Data are shown as Mean ± SEM, *P < 0.05, **P < 0.01, ***P < 0.001, ns indicating *P* > 0.05.

Based on the results of RNA-Seq experiments, 103 differentially expressed genes were identified in the hippocampal tissue of AS-SED and AS-EX mice, with 33 genes being upregulated and 70 genes downregulated (Figure 3A). To visually represent the statistical significance and magnitude of expression changes of these differential genes, we constructed a volcano plot and a heatmap (Figures 3B,C). To further elucidate the potential biological functions of these differentially expressed genes, we conducted Gene Ontology (GO) enrichment analysis and presented the top 10 functional terms, ranked by p-value, in a bubble chart (Figure 3D). This analysis revealed associations of these genes with specific biological processes, molecular functions, or cellular components. Additionally, we identified the top 20 significantly enriched pathway terms and depicted them in a KEGG enrichment bubble chart (Figure 3E). To validate the key genes identified by RNA-Seq analysis, we chose genes from the inflammation-related signaling pathways such as the IL-17 signaling pathway (differential genes: CCL17 and MUC5B), the cAMP signaling pathway (differential genes: EDN1 and HCAR1), the cytosolic DNA-sensing pathway (differential gene: IL-33), and the cytokine-cytokine receptor interaction (differential genes: IL-33 and CCL17). The 5 genes expression data were showed by the heatmap of RNA-Seq (Figure 3F). The qRT-PCR analysis revealed that the IL-33 gene exhibited the most pronounced differential expression among the three experimental groups of mice. Notably, IL-33 expression in the hippocampus was significantly elevated in the AS-SED group relative to the WT group. Conversely, the AS-EX group demonstrated a marked reduction in IL-33 expression compared to the AS-SED group (Figure 3G). These results align with the expression patterns of the IL-33 gene identified in the RNA-Seq data.

### 3.4 Aerobic exercise suppresses the activation of hippocampal IL-33 and NF-κB in AS mice

**FIGURE 4:**
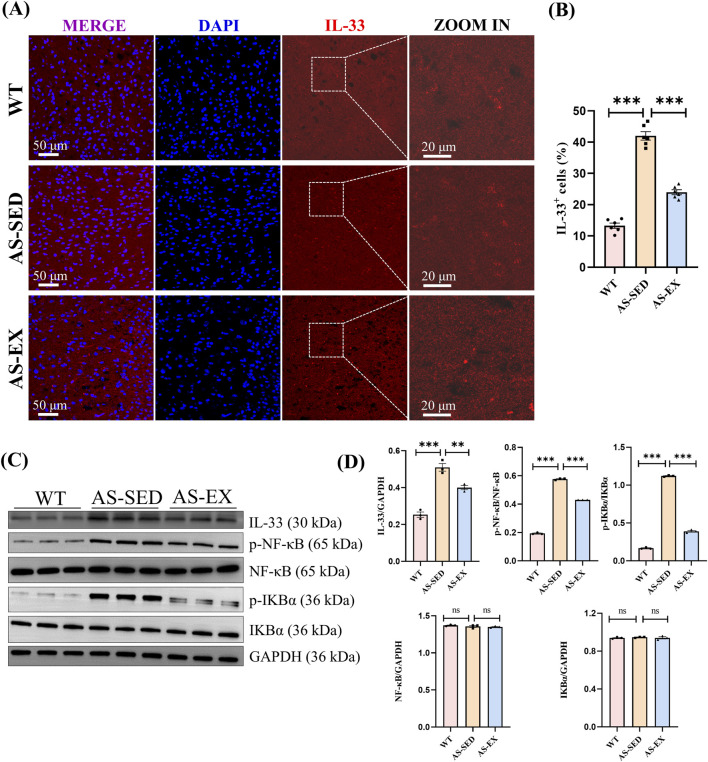
Aerobic exercise reduces IL-33 in the hippocampal tissue and suppresses the activation of NF-κB in AS mice with cognitive impairment. **(A)** Representative immunofluorescence images of IL-33 (red) in the hippocampal region of mice from the three groups (n = 6 per group). **(B)** Quantification of IL-33^+^ cell. **(C)** Immunoblots of IL-33, p-NF-κB, NF-κB, p-IκBα, and IκBα proteins (n = 3 per group). **(D)** Quantification of p-NF-κB/NF-κB and p-IκBα/IκBα ratios and protein expression (n = 3 per group). Data are shown as Mean ± SEM, **P < 0.01, ***P < 0.001, ns indicating *P* > 0.05.

To further explore the potential mechanism of aerobic exercise interventions in reducing IL-33 expression at the cellular level within the hippocampal tissue of AS mice, we employed IL-33 immunofluorescence staining techniques firstly. Our findings revealed a significantly elevated number of IL-33-positive (IL-33^+^) cells in the hippocampal tissue of AS-SED mice compared to the WT group. Conversely, the AS-EX group exhibited a substantial reduction in IL-33^+^ cells relative to the AS-SED group (Figures 4A,B). Moreover, the IL-33 protein also exhibited the same expression trend (Figures 4C,D).These results, when considered alongside previous studies, suggest that aerobic exercise effectively diminishes IL-33 levels in the hippocampal tissue of AS mice.

IL-33 has the capability to trigger the NF-κB signaling cascade (Faas et al., 2021), upregulate numerous pro-inflammatory genes (Mattson, 2005), and is involved in the upregulation of pro-inflammatory cytokines, chemokines, and neurotoxin mediators, such as IL-1β, IL-6, interleukin-8 (IL-8), interferon-gamma (IFN-γ), TNF-α, C-C Motif chemokine ligand 2 (CCL2), glia maturation factor (GMF), nitric oxide (NO), and reactive oxygen species (ROS), which are implicated in the inflammatory processes and cognitive decline characteristic of neurodegenerative diseases (Xiong et al., 2014; Kopitar-Jerala, 2015; Molofsky et al., 2015; Saggu et al., 2016; Ju Hwang et al., 2019). Therefore, WB analyses were performed to assess the expression and phosphorylation levels of key proteins within the NF-κB signaling pathway in the hippocampal tissue of mice across three experimental groups (Figures 4C,D). The findings revealed no statistically significant differences in the protein expression levels of NF-κB and IκBα among the groups. However, relative to the WT group, the AS-SED group exhibited a marked increase in the phosphorylation levels of p-NF-κB/NF-κB and p-IκBα/IκBα, indicating activation of the NF-κB signaling pathway. Conversely, when compared to the AS-SED group, the AS-EX group demonstrated a significant reduction in these phosphorylation levels, suggesting that aerobic exercise intervention effectively attenuates the activation of NF-κB in the hippocampal tissue of AS mice.

### 3.5 Aerobic exercise inhibits the M1 activation of microglia and the release of inflammatory factors in the hippocampal tissue of mice with cognitive impairment induced by AS

**FIGURE 5:**
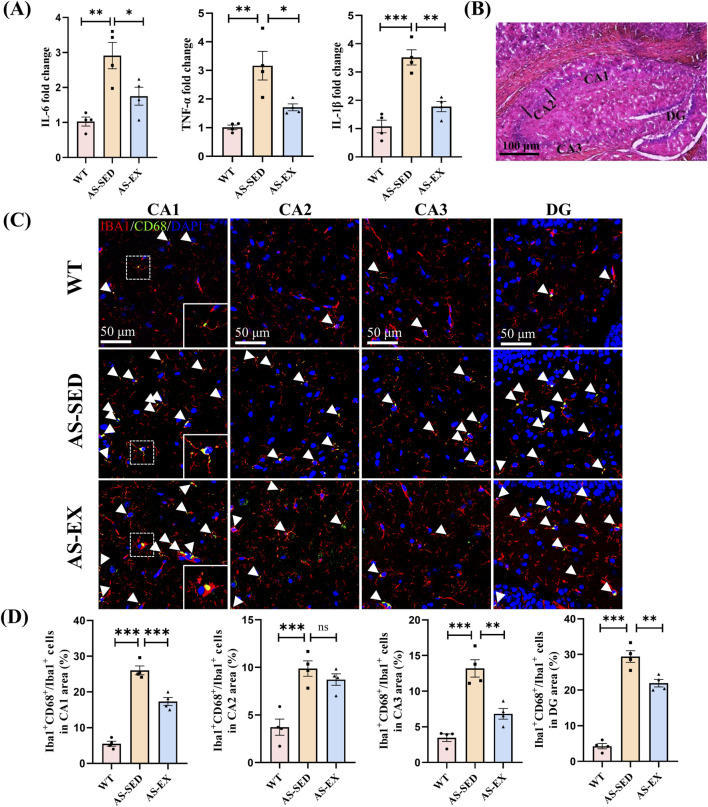
Aerobic exercise suppresses the activation of microglia and the expression of inflammatory factors in the hippocampal CA1, CA3, and DG regions of AS mice. **(A)** Quantitative analysis of inflammatory factors IL-6, TNF-α, and IL-1β in hippocampal tissues from the three groups of mice (n = 4 per group). **(B)** Coronal sections of the mice hippocampus, delineating the CA1 (cornu amoni 1), CA2, CA3, and DG (dentate gyrus) subfields. **(C)** Representative immunofluorescence images of Iba1^+^ (red) and CD68^+^ (green) microglia in the hippocampal CA1, CA2, CA3, and DG regions across three groups of mice. **(D)** Quantification of the percentage of Iba1^+^ and CD68^+^ microglia relative to the total Iba1^+^ microglial population in different hippocampal subfields of the three groups of mice (n = 4 per group). Data are shown as Mean ± SEM, *P < 0.05, **P < 0.01, ***P < 0.001, ns indicating *P* > 0.05.

Aerobic exercise was found to attenuate the expression of inflammatory markers IL-6, TNF-α, and IL-1β in hippocampal tissue (Figure 5A). The data indicated that, relative to WT mice, the levels of IL-6, TNF-α, and IL-1β were significantly elevated in the hippocampus of AS-SED mice, demonstrating a pronounced upregulation of these inflammatory factors in AS mice. In contrast, when compared to the AS-SED group, the expression of these inflammatory markers was markedly reduced in the hippocampus of AS mice subjected to AS-EX, suggesting that aerobic exercise intervention effectively mitigates the expression of inflammatory factors in the hippocampal tissue of AS mice.

Furthermore, Iba1 is expressed in both resting and activated microglia and is widely recognized as a marker for these cells (Ito et al., 2001). CD68 serves as an optimal marker for identifying M1-type activated microglia (Murphy et al., 2005). Consequently, immunofluorescence staining for CD68 and Iba1 was conducted on the hippocampal regions of mice from the three experimental groups, with the results presented in Figures 5B–D. In comparison to the WT group, the AS-SED mice exhibited a significant increase in the percentage of Iba1-positive (Iba1^+^) and CD68-positive (CD68^+^) microglia among Iba1^+^ microglia in the hippocampal CA1 (cornu amoni 1), CA2, CA3, and dentate gyrus (DG) regions. Conversely, when compared to the AS-SED group, the AS-EX mice demonstrated a significant decrease in the percentage of double-positive microglia for Iba1^+^ and CD68^+^ in the hippocampal CA1, CA3, and DG regions. However, no statistically significant difference was observed in the CA2 region between the two groups.

## 4 Discussion

The primary aim of this study is to investigate the ameliorative effects of aerobic exercise on cognitive impairments induced by AS and to explore its underlying mechanisms. ApoE^−/−^ mice fed a HFHCD have been established as a common model for AS in mice (Ilyas et al., 2022). To determine whether ApoE^−/−^ mice developed significant arterial plaques after 6 weeks of HFHCD feeding, we compared them with C57BL/6J mice fed a normal diet to assess plaque area, a methodology consistent with previous studies (Li L. et al., 2019). Recent research has demonstrated that ApoE^−/−^ mice fed an HFHCD to establish an AS model develop cognitive impairments (Sun et al., 2024). Our findings corroborate these observations, as significant atherosclerotic plaque formation was detected in the aortas of AS mice compared to WT mice after 6 weeks on an HFHCD. Furthermore, AS mice exhibited significantly poorer performance in the Y-maze, open-field test, and three-chamber social test compared to WT mice. These results validate the efficacy of our model in inducing AS-related cognitive impairment.

Exercise has been validated in both human and animal studies to improve AS, with systematic reviews focusing on the mechanisms by which aerobic and anaerobic exercises ameliorate AS (Aengevaeren et al., 2020; Aengevaeren et al., 2023; Liu et al., 2023; Yang et al., 2023). However, current research on AS improvement has primarily concentrated on cardiovascular function, with scant attention given to cognitive impairments. Exercise has been shown to improve cognitive function in COVID-19 patients, elderly with mild cognitive impairment, and patients with dementia and Alzheimer’s disease (Barker-Davies et al., 2020; Tomoto et al., 2021; Sun et al., 2023). In our study, a 10-week aerobic exercise intervention resulted in a marked decrease in aortic plaque area in AS mice subjected to exercise. Additionally, these exercised AS mice demonstrated significantly superior performance in behavioral tests compared to their sedentary counterparts. These results suggest that a 10-week regimen of aerobic exercise not only diminishes atherosclerotic plaque burden but also enhances cognitive abilities in AS mice.

AS can result in inadequate cerebral perfusion and oxygen utilization, potentially leading to cognitive decline (Li B. et al., 2019). Previous animal studies have indicated that exercise may enhance cerebral blood flow in mice with vascular cognitive impairment and dementia (Khan et al., 2024). In this study, we employed LSCI to assess cerebral blood flow across three groups of mice. However, due to the technical constraints of LSCI, our observations were limited to the blood flow in the superficial veins of the cerebral cortex. We quantified cortical cerebral blood flow at the sagittal suture of the mouse brain and observed that aerobic exercise ameliorated the reduced cerebral blood flow associated with AS. Future research should aim to investigate changes in deep brain blood flow to ascertain which specific cerebral vessels exhibit improved blood flow as a result of aerobic exercise.

AS is recognized as a chronic inflammatory condition, with one potential mechanism contributing to cognitive impairment being brain inflammation (Schreyer et al., 1998; Lama et al., 2022). Considering this association and the role of the hippocampus as a central hub for cognitive functions such as learning and memory (Benarroch, 2013),we conducted RNA-seq, qRT-PCR, and immunofluorescence staining for IL-33 on hippocampal tissues from AS-SED and AS-EX groups. Our research focused on the target gene IL-33, which plays a role in inflammatory signaling pathways. Identified in 2005 as a novel member of the interleukin-1 cytokine family, IL-33, also known as IL-1F11, can function as a pro-inflammatory cytokine when released in excess (Schmitz et al., 2005). It activates inflammatory cells in the central nervous system, including astrocytes, microglia, and mast cells (Rao et al., 2022). The NF-κB signaling pathway is activated by IL-33, which promotes the upregulation of various pro-inflammatory genes (Mattson, 2005). This process enhances the production of inflammatory mediators such as IL-1β, IL-6, IFN-γ, TNF-α, CCL2, GMF, NO, and ROS, ultimately driving inflammatory responses and cognitive decline in neurodegenerative diseases (Xiong et al., 2014; Kopitar-Jerala, 2015; Molofsky et al., 2015; Saggu et al., 2016; Ju Hwang et al., 2019). Our research demonstrates that HFHCD induces elevated expression levels of IL-33 in the hippocampal tissues of AS model mice. Notably, aerobic exercise markedly mitigates this overexpression. Western blot analyses further reveal that aerobic exercise suppresses the phosphorylation of NF-κB and IκBα within the hippocampal region of AS mice, thereby attenuating the hyperactivation of NF-κB.

**FIGURE 6:**
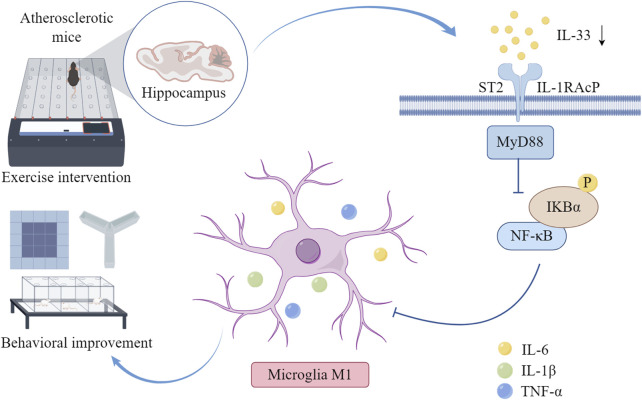
Aerobic exercise can alleviate neuroinflammation and improve cognitive function reducing hippocampal IL-33 and NF-κB activation in AS mice, thereby reducing the M1-type activation of microglia and the release of inflammatory factors IL-6, IL-1β, and TNF-α in the hippocampal tissue of AS mice. The diagram was drawn by Figdraw (www.figdraw.com).

Microglia, widely dispersed across the brain, function as the key innate immune cells and are the first to react to pathological injuries (Baufeld et al., 2018). In the central nervous system, their role can shift between promoting inflammation and providing neuroprotection, depending on their activation status (Tang and Le, 2016). When triggered by pro-inflammatory cytokines from pathogens or injured cells, resting microglia are activated to release inflammatory mediators like IL-1β, TNF-α, IL-6, and NO. These factors are harmful in neurodegenerative conditions and are associated with the M1 phenotype (Liddelow and Barres, 2017). This neuroinflammatory response contributes to cognitive impairment (Wilson et al., 2002). Studies have shown that IL-33 promotes the release of pro-inflammatory mediators and neurotoxic substances from activated microglia and astrocytes *in vitro* (Cao et al., 2018). This scenario establishes a self-perpetuating cycle; for instance, pro-inflammatory mediators such as IL-1β, TNF-α, GMF, ROS, NO, and CCL2, produced by IL-33-activated microglia, subsequently activate additional microglia to secrete further pro-inflammatory mediators and elevate IL-33 secretion from astrocytes and microglia (Rao et al., 2022). Numerous studies have indicated that physical exercise can attenuate the release of inflammatory factors in the brain and inhibit the M1 activation of microglia (Mee-Inta et al., 2019; Liu et al., 2024). Our study aligns with prior research, demonstrating that aerobic exercise suppresses the activation of M1 phenotype microglia in the hippocampus of mice with AS-induced cognitive impairment and reduces the expression of inflammatory cytokines IL-6, TNF-α, and IL-1β. Notably, exercise did not attenuate M1 microglial activation in the CA2 region of the hippocampus in AS mice, potentially due to a limited sample size or other factors, warranting further investigation. Overall, our findings suggest that aerobic exercise may ameliorate neuroinflammation in AS mice, thereby enhancing cognitive function (Figure 6).

## 5 Conclusion

To sum up, our research shows that a 10-week program of aerobic exercise successfully reduces the excessive expression of IL-33 in the hippocampus of AS mice and curbs the overactivity of the NF-κB pathway. This, in turn, reduces M1 microglial activation and the release of hippocampal inflammatory cytokines IL-6, TNF-α, and IL-1β, ultimately enhancing cognitive function in AS mice. These findings offer preliminary yet compelling evidence supporting the role of exercise in ameliorating cognitive impairment in AS patients and address a critical gap in the understanding of exercise as a preventative measure against AS-induced cognitive deficits, thereby laying the groundwork for future research in this domain.

## Data Availability

RNA-seq data have been deposited at NCBI BioProject, accession number: PRJNA1195224.
